# Primary Cardiac Sarcoma: A Rare, Aggressive Malignancy with a High Propensity for Brain Metastases

**DOI:** 10.1155/2019/1960593

**Published:** 2019-03-10

**Authors:** Brittany L. Siontis, Lili Zhao, Monika Leja, Jonathan B. McHugh, Maryann M. Shango, Laurence H. Baker, Scott M. Schuetze, Rashmi Chugh

**Affiliations:** ^1^Department of Internal Medicine, University of Michigan, 1500 E Medical Center Drive, Ann Arbor, MI 48109, USA; ^2^Department of Medical Oncology, Mayo Clinic, 200 First Street SW, Rochester, MN 55901, USA; ^3^Department of Biostatistics, University of Michigan, 1415 Washington Heights M2541, SPHII, Ann Arbor, MI 48109, USA; ^4^Department of Pathology, University of Michigan, 1500 E Medical Center Drive, Ann Arbor, MI 48109, USA; ^5^Swedish Medical Center Cancer Institute, 21632 Highway 99, Edmonds, WA 98026, USA

## Abstract

**Introduction:**

Primary cardiac sarcoma (PCS) has a poor prognosis compared to other sarcomas due to late presentation, challenging resection, incidence of metastases, and limited efficacy of systemic therapies.

**Methods:**

A medical record search engine was queried to identify patients diagnosed with PCS from 1992 to 2017 at the University of Michigan.

**Results:**

Thirty-nine patients with PCS had a median age of 41 years (range 2–77). Common histologies were angiosarcoma (AS, 14), high-grade undifferentiated pleomorphic sarcoma (UPS, 10), and leiomyosarcoma (LMS, 5). Sites of origin were left atrium (18), right atrium (16), and pericardium (5). AS was the most common right-sided tumor; UPS was more common on the left. Eighteen patients presented with metastases involving lung (10), bone (7), liver (5), and brain (4). Twenty-five patients underwent resection, achieving 3 R_0_ resections. Patients received a median of 2 (1–6) systemic therapies. Median overall survival (OS) was 12.1 months (range 0–79). Median OS was 14.0 months and 8.2 months in patients who did or did not undergo resection, respectively (*p*=0.018). Brain metastases occurred in 12 (31%) patients, 9 (75%) of whom had left heart tumors, at a median of 8.5 months (range 0–75) from diagnosis. Median OS was 5.6 months (range 0–30) after the diagnosis of brain metastases.

**Conclusions:**

PCS portends a poor prognosis, because of difficulty in obtaining complete resection of sarcoma, advanced stage at diagnosis, and high risk of brain metastases. Providers should be aware of the increased risk of brain metastases and consider brain imaging at diagnosis and follow-up.

## 1. Introduction

Primary cardiac tumors are rare, with an overall incidence of 0.001–0.02% [1]. Of these, 25% are malignant, most commonly sarcomas. Consequently, the management of patients with primary cardiac sarcomas (PCSs) is extrapolated from small case series of afflicted patients and larger series of patients with sarcomas of different sites of origin but similar histologic subtypes. PCSs are distinct entities with many unique features coincident with their site of origin. As tumors are deep, visceral, and involve a critical organ, disease is often locally advanced impairing resection by the time symptoms develop.

We present a case series of 39 patients with PCS in attempt to characterize and better understand disease presentation, location, treatment modalities, and overall survival of patients with PCS. We highlight patterns of metastatic disease, histologic subtypes, and the barriers to improvements in overall survival of PCS patients.

## 2. Materials and Methods

### 2.1. Patient Identification

The University of Michigan Electronic Medical Record Search Engine (EMERSE) was searched using the terms “cardiac sarcoma” and “heart” plus “sarcoma” to identify patients with a diagnosis of primary cardiac sarcoma treated at our institution between 1992 and 2017. Patients with sarcomas originating within the pulmonary vasculature or great vessels were not included in this analysis. Details regarding demographics, clinical presentation, pathologic features, treatment protocols, and outcome were extracted from clinical records. All research was approved by the University of Michigan Institutional Review Board (HUM00068553).

### 2.2. Statistical Analysis

Descriptive statistics, such as median and range, were calculated for continuous variables, and frequencies were presented for categorical variables. To compare two categorical variables, a frequency table was created and analyzed using the chi-squared test or Fisher's exact test. Overall survival (OS) was estimated by the Kaplan–Meier method and compared using the log-rank test. Statistical significance was defined as a two-sided *p* value <0.05. All analyses were conducted using SAS (version 9.4, SAS Institute, Cary, NC).

## 3. Results

### 3.1. Demographics and Disease Characteristics

Thirty-nine patients with PCS presenting to the University of Michigan between 1992 and 2017 were identified. Median age at diagnosis was 41 years. Patient demographics and brief outline of disease characteristics are summarized in Table 1. The most common site of origin was the left heart.

All patients were symptomatic at time of diagnosis, most frequently with dyspnea (*n*=29), chest pain (*n*=8), cough (*n*=3), bilateral lower extremity edema (*n*=3), and hemoptysis (*n*=3). Twenty patients had evidence of pericardial effusion at diagnosis, including all 5 patients with pericardial tumors and 11/16 patients with right-sided tumors. Twelve patients had evidence of tamponade at diagnosis, one of which had a left atrium (LA) tumor, 8 had right atrium (RA) tumors, and 3 had pericardial tumors. Eight patients had pulmonary hypertension at time of diagnosis. There were no clinically significant arrhythmias present at diagnosis.

**Figure 1:**
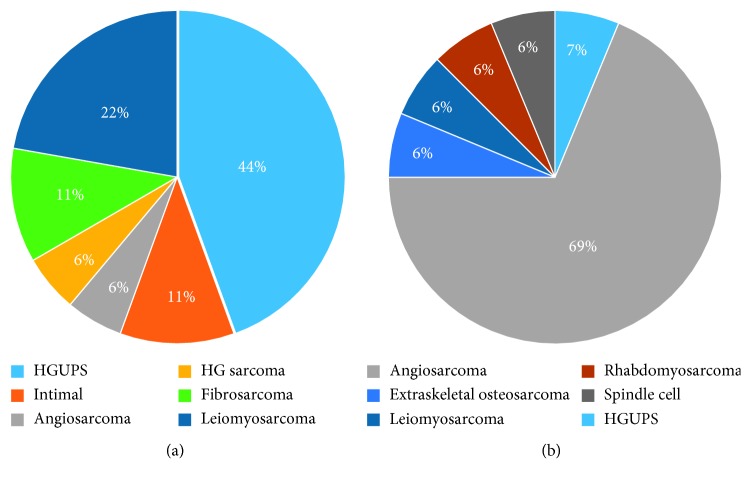
Primary cardiac histology by tumor location.

Angiosarcoma was the most common tumor histology and was more commonly located on the right side of the heart (*n*=11) and pericardium (*n*=2, *p* < 0.0001) compared to other histologies. UPS (*n*=8) and LMS (*n*=4) were the most commonly observed left-sided tumors (Figure 1).

### 3.2. Metastases

Metastatic disease was present in 18/39 (46%) of PCS patients at the time of diagnosis. The distribution of metastases at the time of disease diagnosis is listed in Table 1.

Twelve patients developed brain metastases, 4 of whom had brain metastases at initial diagnosis. All presented with neurologic symptoms. Median time from diagnosis of PCS to diagnosis of brain metastases was 8.5 months (range 0–75.8 months). Left-sided cardiac tumors were more commonly associated with brain metastases (9/12, *p*=0.01) compared to right-sided and pericardial tumors. The most common histological variant was UPS (*n*=6). Other histological types included angiosarcoma (3), leiomyosarcoma (2), and fibrosarcoma (1). All patients with right-sided tumors and brain metastases at diagnosis (*n*=3) also had lung metastases. One patient had evidence of a right to left shunt on echocardiogram as detected by a bubble study.

### 3.3. Surgery

Twenty-five patients (64%) underwent surgical resection of their PCS. Eleven of the twenty-five (11/25, 44%) patients had evidence of metastatic disease at diagnosis. An R_0_ resection was obtained in only 3 patients (12%), all of whom had left atrial tumors. Nine patients had R_1_ resections, 11 patients had R_2_ resections, and the surgical margin in 2 patients was unknown. Five patients with solitary brain metastases underwent craniotomy as primary management of their metastatic disease.

### 3.4. Radiation Therapy

In total, 11 patients (26%) received radiation therapy. Three patients had radiation to the primary cardiac tumor, one to alleviate superior vena cava syndrome and two to optimize clinical status prior to systemic chemotherapy. Eight patients received radiation therapy to brain metastases (5 whole brain and 3 stereotactic radiotherapy). Four patients received radiation therapy after resection of solitary brain metastases. Four patients received radiation therapy alone for multifocal brain metastases. Two of these patients received palliative radiation to symptomatic lesions in the bone and lung.

### 3.5. Systemic Therapy

**Figure 2:**
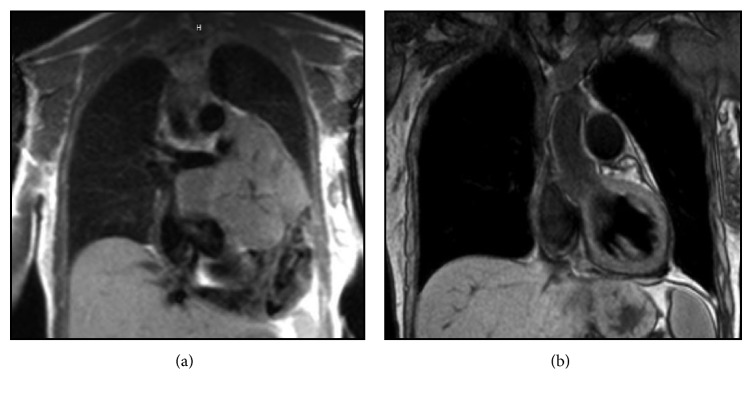
Baseline cardiac MRI shows a large, heterogeneous mass in the pericardial space with a large effusion and compression of the left atrium (a). Neoadjuvant chemotherapy with doxorubicin/ifosfamide ×12 cycles and gemcitabine/docetaxel ×6 cycles resulted in near complete response (b).

Twenty-eight patients (72%) received chemotherapy, with a median of 2 regimens (range 1–6). Twenty-one patients (75%) who received chemotherapy had resection of their primary tumor. Two patients received neoadjuvant chemotherapy followed by resection, R_0_, and R_1_.  Figure 2 shows baseline (a) and posttreatment (b) imaging for a patient who received neoadjuvant chemotherapy with good response, enabling subsequent resection.

Most patients received systemic therapy with palliative intent. Seventeen patients (61%) received doxorubicin plus ifosfamide (AI) as first-line therapy with a mean of 5 cycles (range 0.5–9). Ten patients received gemcitabine and docetaxel (GT) as first (*n*=2) or second-line (*n*=8) therapy. Eight patients received 3 or more lines of therapy. Other chemotherapy regimens frequently used in any line included paclitaxel (*n*=5), dacarbazine (*n*=3), and ifosfamide (*n*=3).

### 3.6. Survival

**Figure 3:**
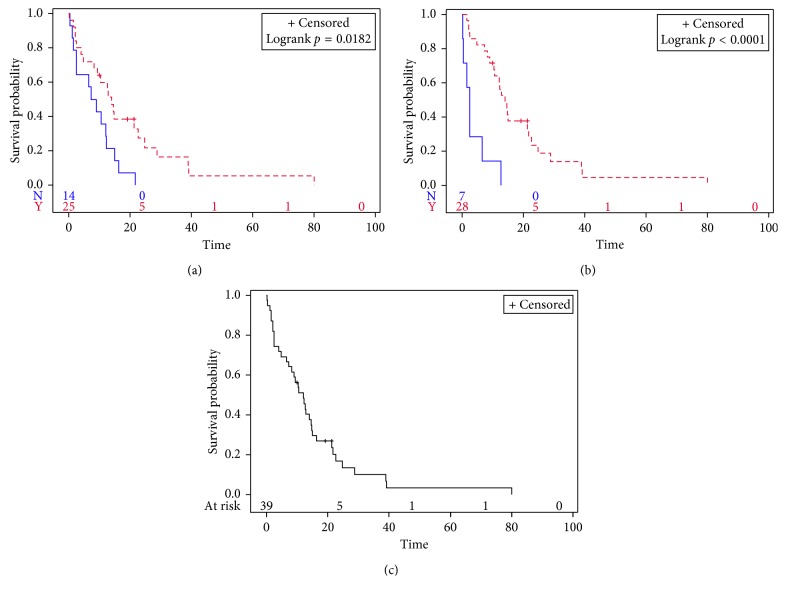
Overall Survival of cardiac sarcoma patients (*n*=39). Overall survival was significantly improved with surgical resection of PCS (a) and chemotherapy administration (b). For all patients (c), median overall survival was 12.1 months (range 0–79 months). The dashed curve represents surgery or chemotherapy and solid curve no intervention.

The median OS for patients who underwent surgery was 14.0 months (range 1–79 months), compared to 8.2 months (range 0–33 months; *p*=0.02) for patients who did not (Figure 3(a)). Patients who received chemotherapy lived longer compared to patients who did not with a median OS of 14.0 months versus 2.4 months, respectively (*p*=0.0001, Figure 3(b)). Median OS for all patients was 12.1 months (range 0–79 months; Figure 3(c)). Three patients were alive at the time of data assessment in October 2017, one diagnosed in 2015 and two diagnosed in 2016 (median follow-up time of 19 months). Median time from diagnosis of brain metastases to death was 5.6 months (range 0–30 months).

Survival differences were not statistically significant based on the presence of metastatic disease at diagnosis, tumor location, or tumor histology because, in part, of our small sample size.

## 4. Discussion

We present our single-institution experience with primary cardiac sarcoma with several notable findings: a high incidence of metastatic disease to the brain as compared to sarcomas originating in other locations, pattern of metastatic spread associated with location of sarcoma origin within the heart, predilection of certain histologies to originate in the right versus left heart, and association of multimodality therapy with improved patient survival despite the lack of a standardized treatment approach.

We found brain metastases occurred quickly and frequently in our PCS patients. Thirty-one percent (12/39) of our PCS patients developed brain metastases, compared to 1–8% in noncardiac sarcomas [2–7]. This is close to the estimated 10–30% prevalence of brain metastases in all cancer patients [8]. The median length of time from diagnosis to the development of brain metastases has been reported to be about 27 months in other soft tissue sarcomas [2, 3, 6, 9, 10]. In our patient cohort, however, the median time from diagnosis of PCS to brain metastasis was 8.5 months (range 0.3–75.8 months). This may be secondary to the intracardiac location of primary tumors, potentially providing a more direct conduit from tumor to the brain via the cardiac outflow tract, particularly in left-sided tumors. There are case reports of brain metastases developing in patients with angiosarcoma, HGPUS, and leiomyosarcoma [3–5, 7, 9–16]. In our series, the most common histology to metastasize to the brain was high-grade undifferentiated pleomorphic sarcoma (*n*=6).

The prevalence of brain metastases in sarcoma is increasing due to prolonged survival from multimodal therapies that have poor CNS penetration [17, 18]. In our patients who lived longer than 12 months (22 pts), brain metastases eventually developed in 9 (41%). Median OS for our patients with brain metastases was 13.6 months, with a post-brain metastases median survival of 5.6 months. This is significantly shorter than reports of brain metastases in sarcomas of all primary sites, which have a median of 13.9 months from diagnosis to development of brain metastases and overall and postmetastasis survival of 15 and 9.2 months, respectively [3, 5].

The management of brain metastases is largely dependent on the number of lesions and patient performance status. Surgical intervention is considered with solitary metastases, while radiation therapy is preferred for multifocal disease or in patients with marginal performance status. Metastasectomy of brain metastases is reported to result in increased post-brain metastases survival to about 9 months, compared with 2.7 months for those who did not receive surgical intervention [3]. In our cohort, one patient underwent metastasectomy alone with a post-brain metastases survival of 12 months. In our cohort, 4 patients received radiation therapy for brain metastases with a median post-brain metastases survival of 8 months. Four patients underwent surgical resection followed by radiation with a post-brain metastases survival of 5.5 months. Since most patients had received chemotherapy by the time they develop brain metastases, the role of chemotherapy in brain metastases remain unclear [5, 16].

Surgical resection is the preferred management for localized sarcomas. As compared to other sarcomas, PCS patients are more likely to have advanced stage or inoperable primary tumor at presentation. Barriers to complete resection include anatomic location and declining performance status due to tumor at presentation. Given the difficulty in achieving complete resection with cardiac tumors, autotransplantation has been explored to allow for complete resection and optimal reconstruction. Ramlawi et al. reported on their institutional experience with cardiac transplant in 34 patients with left-sided cardiac tumors [19]. In their series, 26 patients had primary cardiac sarcomas. In those with malignant tumors, 1- and 2-year survival was 46% and 28%, respectively. While the authors conclude cardiac autotransplantation to be feasible and safe for complex left-sided tumors, this should only be considered in certain individuals and performed at experienced specialty centers.

Nearly 50% of our patients had evidence of metastatic disease at time of presentation, which is higher than the reported 20–30% in several case series [20–23]. In our patient cohort, metastatic disease at presentation did not impact survival compared with those with localized disease (14 months versus 10 months, *p*=0.12, respectively), while surgical resection did (*p*=0.02), highlighting the importance of local disease control. This survival benefit of resection has been noted in other reports of PCS and is independent of the presence of metastatic disease [24]. This may be due to the preservation of cardiac function as heart failure has been described as the most common cause of death of PCS patients [25]. Furthermore, improved cardiac function ultimately aids in ability to administer systemic therapy, specifically an anthracycline.

Surgery alone, however, is not enough to prevent primary tumor recurrence or distant metastases. Most patients who undergo complete (R_0_) resections without metastatic disease will relapse, highlighting the need for systemic therapy [22, 26]. Although multimodal therapy for PCS patients is associated with improved survival [20, 21, 26], the specific role of radiation and chemotherapy alone or in combination in PCS has yet to be defined. A univariate analysis by Isambert et al. found that chemotherapy offered a survival advantage only in patients who did not undergo primary cardiac tumor resection [24]. Abu Saleh et al. reviewed the utility of neoadjuvant chemotherapy in the management of right-sided cardiac tumors in 44 patients [27]. In their cohort, 73% of patients received neoadjuvant chemotherapy consisting of doxorubicin and ifosfamide. There was a significant improvement in median overall survival in patients who achieved a complete (R_0_) versus R_1_ resection, 53.5 months and 9.5 months, and those who received neoadjuvant chemotherapy versus primary surgery, 20 versus 9.5 months, respectively. The authors conclude that neoadjuvant chemotherapy improves the ability to achieve complete resection and thus translates into improved survival. However, the ability to deliver neoadjuvant chemotherapy relies on accurate preresection diagnosis. Given the rarity of these tumors and clear benefits to multimodality therapies, it is imperative to employ a multidisciplinary team approach. The creation of a multispecialty, multi-institution cardiac tumor board in which our institution participates enhances the ability for cross-discipline discussions to optimally manage these challenging tumors.

In our patient cohort, those who received chemotherapy alone and those who underwent surgical resection and received systemic chemotherapy had a survival advantage (*p*=0.039) compared to those who did not receive chemotherapy. Given the retrospective nature of this study, it is important to note that patients who receive chemotherapy generally have better performance status and organ function, which likely impacted the survival advantage noted with chemotherapy. Local disease control, resulting in improved cardiac function, likely results in better performance status and ability to tolerate aggressive therapies.

## 5. Conclusions

Primary cardiac sarcomas are rare, confer increased risk of brain metastases, are associated with shorter survival duration, and have a higher disease-specific mortality compared to soft tissue sarcomas arising in extracardiac sites. Increased mortality may be due to the difficulty with complete resection and high disease and symptom burden at presentation, making aggressive multimodal therapy difficult. Multidisciplinary care incorporating surgery, radiation, and systemic therapy when appropriate constitutes ideal management. Based on our findings, we recommend brain imaging at the time of primary cardiac sarcoma diagnosis given the incidence of brain metastases. Furthermore, our results suggest a role for aggressive multimodal therapy including resection and systemic therapy, even in the metastatic setting, to improve outcomes.

## Figures and Tables

**Table 1:** Patient demographics and disease characteristics.

	No. of patients (%)
Male	13 (33)
Female	26 (67)
Median tumor size in cm (range)	6.5 (1.7–16)
Median no. of total chemotherapy regimens	2 (range 1–6)
Primary tumor location	
Right heart	16 (41)
Left heart	18 (46)
Pericardium	5 (13)
Histology	
Angiosarcoma	14 (36)
High-grade undifferentiated pleomorphic sarcoma	10 (26)
Leiomyosarcoma	5 (13)
Intimal sarcoma	3 (8)
Fibrosarcoma	2 (4)
Other	5 (13)
Metastatic disease present at diagnosis	18 (46)
Sites of metastases	
Lung	10 (55)
Bone	7 (39)
Liver	5 (28)
Brain	4 (22)
Pancreas	1 (5)
Adrenal	1 (5)
Treatment	
Surgery	25 (58)
R_0_	3 (12)
R_1_	9 (36)
R_2_	11 (44)
Unknown	2 (8)
Chemotherapy	28 (72)
Radiation to cardiac tumor	3 (8)
Median age in years (range)	41 (2–77)

## Data Availability

The data sets generated and analyzed to support the findings of this study may be available from the corresponding author upon request.
